# Pressure-Induced Polymorphism of Caprolactam: A Neutron Diffraction Study

**DOI:** 10.3390/molecules24112174

**Published:** 2019-06-10

**Authors:** Ian B. Hutchison, Craig L. Bull, William G. Marshall, Andrew J. Urquhart, Iain D.H. Oswald

**Affiliations:** 1 Strathclyde Institute of Pharmacy & Biomedical Sciences (SIPBS), University of Strathclyde, 161 Cathedral Street, Glasgow G4 0RE, UK; ian.b.hutchison@gmail.com; 2 ISIS Neutron and Muon Source, Science and Technology Facilities Council, Rutherford Appleton Laboratory, Harwell, Didcot OX11 0QX, UK; craig.bull@stfc.ac.uk; 3 Department of Health Technology, Technical University of Denmark, Produktionstorvet, 2800 Kgs. Lyngby, Denmark; anur@dtu.dk

**Keywords:** high-pressure single-crystal X-ray diffraction, high-pressure neutron diffraction, phase transitions, intermolecular interactions, energy frameworks

## Abstract

Caprolactam, a precursor to nylon-6 has been investigated as part of our studies into the polymerization of materials at high pressure. Single-crystal X-ray and neutron powder diffraction data have been used to explore the high-pressure phase behavior of caprolactam; two new high pressure solid forms were observed. The transition between each of the forms requires a substantial rearrangement of the molecules and we observe that the kinetic barrier to the conversion can aid retention of phases beyond their region of stability. Form II of caprolactam shows a small pressure region of stability between 0.5 GPa and 0.9 GPa with Form III being stable from 0.9 GPa to 5.4 GPa. The two high-pressure forms have a catemeric hydrogen-bonding pattern compared with the dimer interaction observed in ambient pressure Form I. The interaction between the chains has a marked effect on the directions of maximal compressibility in the structure. Neither of the high-pressure forms can be recovered to ambient pressure and there is no evidence of any polymerization occurring.

## 1. Introduction

Nylon-6 is one of the most versatile polymers known due to its physicochemical properties, e.g. toughness and stiffness. These properties make it an ideal polymer for use in a number of applications ranging from healthcare, e.g. toothbrush bristles and sutures, to everyday objects such as clothing and strings for musical instruments. The ability of the polymer to cause a hydrogen bond provides a degree of crystallinity that affects the overall properties of the polymer [1]. There are a number of different polymorphs of nylon-6 but the main ones are the α- and γ-forms of which the α-form is the most thermodynamically stable, whilst the γ-form is the kinetically observed structure. The two different polymorphs differ in the relative orientation of the polyamide chains. The chains form an anti-parallel arrangement in the α-form whilst they are parallel in the γ-form. Additionally, the γ-form has additional torsional rotations, making the chains less linear than the α-form. Conversion from α- to the γ-form can be performed via heating, solvents (through annealing process) or induced by strain through the mobility of the chain structures. The polymerization used to synthesize nylon-6 is a ring-opening polymerization of caprolactam. Nylon-6 is typically synthesized via ring-opening of caprolactam (C_6_H_11_NO) in an inert nitrogen environment at temperatures in excess of 500 K although synthesis can be performed at lower temperatures (150 K) using anionic polymerization [2]. 

There have been a number of studies that have investigated solid-state reactions either through photo-induced methodologies [3,4] or through the use of the pressure [5,6,7]. Acetylene and its derivatives [8,9,10,11,12,13], benzene [10] and even simple molecules such as carbon dioxide [14,15] have been polymerized in the solid state using pressure. Reactions in the solid state facilitate the production of a polymer with a particular architecture since the molecules are set into a certain arrangement by the crystal structure before the reaction occurs [16,17]. We have previously investigated the cyclic di-ester, glycolide, as it is the pre-cursor to poly-glycolic acid, a significant polymer for pharmaceutical applications [18]. In this system, we observed polymorphism with respect to pressure; however, there was no indication that the polymerization had taken place to the maximum pressure of 6 GPa [19,20]. Given this result, we investigated ε-caprolactam under high-pressure conditions. 

Caprolactam is the cyclic amide precursor to Nylon-6 and is a highly crystalline colourless material. Oya and Myasnikova first reported the crystal structure of ε-caprolactam crystallised from a heptane:benzene solution [21]. They assigned the monoclinic *B*1 1 2/*b* space group to the structure and reported that the molecule adopted a chair conformation with the amide group being planar (Cambridge Structural Database (CSD) [22] refcode: CAPLAC01). Their choice of cell was based on a previous determination of the space group being *Ic* or *I*2/*c* by Assarson; however, no reasoning was given behind their choice of non-standard setting [23]. Winkler and Dunitz followed this report with a redetermination of the crystal structure of ε-caprolactam under ambient conditions in the standard monoclinic setting of *C*2/*c* (CAPLAC) [24]. 

In this paper, we report the highly polymorphic behavior of caprolactam with respect to pressure and the importance of kinetics in the observation of the two high-pressure phases using a combination of single-crystal X-ray and neutron powder diffraction. In addition, we have re-determined the structure of ε-caprolactam at 123 K. From the experimental data, we performed calculations using CrystalExplorer [25] to provide an energetic description of the three polymorphs of ε-caprolactam and to address the observed behavior at higher pressures.

## 2. Results

### 2.1. Caprolactam Form I Crystal Structure

**Figure 1:**
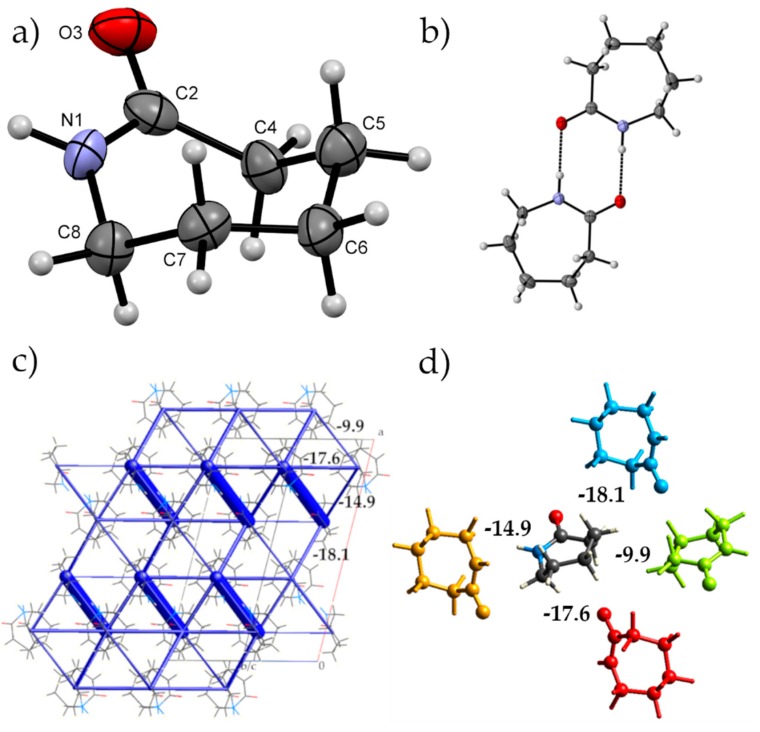
(**a**) Numbering scheme for ε-caprolactam highlighting the chair conformation that the molecule adopts in the crystal structure under ambient conditions (**b**) The amide dimer hydrogen-bonding interaction between two ε-caprolactam molecules that is observed in the crystal structure (**c**) The total interaction energy framework of ε-caprolactam as calculated by CrystalExplorer [25]. The thickness of the framework indicates the strength of interaction (dimer interaction: −68.2 kJ·mol^−1^). Other interaction energies between the dimers are indicated. (**d**) The top four non-hydrogen bonding interactions of the central molecule with nearest neighbours (values are in kJ·mol^−1^).

We will first describe the ambient-pressure crystal structure of ε-caprolactam before describing the high pressure behaviour using energies calculated by CrystalExplorer. Using this methodology, the energies between molecules in the crystal structure can be calculated and partitioned into coulombic, dispersion, polarisation and repulsion so that a more in-depth understanding can be made of the interactions between molecules and their hierarchy. In the following manuscript, we will be using energies derived from the geometry optimized structures. The dominant feature of the packing arrangement is an amide dimer interaction between two of the adjacent caprolactam molecules (Figure 1b,c, N…O 2.8754(12) Å; intermolecular energy of –68.2 kJ·mol^−1^). The dominance of this interaction in the crystal structure is clearly visible using the energy frameworks calculated by CrystalExplorer. Infrared (IR) investigations of caprolactam in carbon tetrachloride have shown that in solution there is a propensity for the molecules to form dimers; hence, it fits that this is observed in the crystal structure [26]. These dimers interact through close contact between H2B and H6B and O1 that connects dimers along the {0 −1 1} directions enabled by the perpendicular arrangement of neighbouring dimers (−17.6 kJ·mol^−1^; Figure 1d). These interactions have a large electrostatic component and are located within a layer around *a* = ¼, ¾ etc. The other interactions within these layers show a more dispersive interaction, for example, the orange molecule in Figure 1d (−14.9 kJ·mol^−1^) where the molecules interact in an edge-to-face manner between H6A of the central molecule and the carbonyl group. Interaction between the layers is through an edge-to-edge interaction (H3A…O1 close contact, blue molecule; −18.1 kJ·mol^−1^) that is largely dispersive in nature. The close packing around the central molecule is completed by various van der Waals interactions with molecules located around the organic part of the molecule typified by the light green interaction in Figure 1d. Here, the neighbouring dimers are interacting end-on with one another. 

### 2.2. Diamond Anvil Cell Experiments

We initially investigated caprolactam using single-crystal X-ray diffraction using a crystal grown from an evaporative crystallisation using ethyl acetate as the solvent. We were able to collect two datasets at high pressure (0.64 and 1.64 GPa) before the sample lost crystallinity at 2.31 GPa. At both 0.64 and 1.64 GPa, the crystal was indexed as the known form, albeit with compressed cell axes. On downloading, we did observe that the crystal had become soft rather than the brittle nature of the crystals from the crystallisation experiment, indicating that the solid had been modified in some way. To verify this, we collected data on another crystal that was harvested from a vial of 1-butanol. The choice of crystal from 1-butanol was based on the higher quality crystals grown from this solvent. Initial indexing confirmed that the crystal was the known form of caprolactam before we loaded the sample into the diamond anvil cell. We collected datasets at 1.20 GPa, 1.70 GPa and 2.18 GPa (the latter pressure was taken for comparison of unit cell parameters with the neutron data). At the first pressure point (1.20 GPa), we noted that the diffraction was of lower quality (multiple weaker crystals) than we observed from the unit cell check at ambient pressure (strong single crystal diffraction) and that the crystal indexed as a new form. The crystal had undergone a reconstructive phase transition on application of pressure; however, the fragmentation of the crystal still allowed data reduction and analysis of the crystal structure. This form has been designated as Form III due to subsequent analysis of all the data from the study and to ensure the nomenclature is consistent with the phase diagram and not due to time of discovery (Table S1). 

The change in behaviour depending on the solvent of recrystallization was an interesting phenomenon; hence, we used neutron powder diffraction to investigate the phase behaviour of caprolactam at pressure and with respect to the solvent of recrystallization. Powder diffraction would enable us to follow the transitions without the reconstructive transition causing issues during data reduction i.e. the fracture of the single crystal. The larger volumes permitted by the Paris-Edinburgh press provide a much better powder averaging and representation of the bulk.

### 2.3. Neutron Powder Diffraction Studies

**Figure 2:**
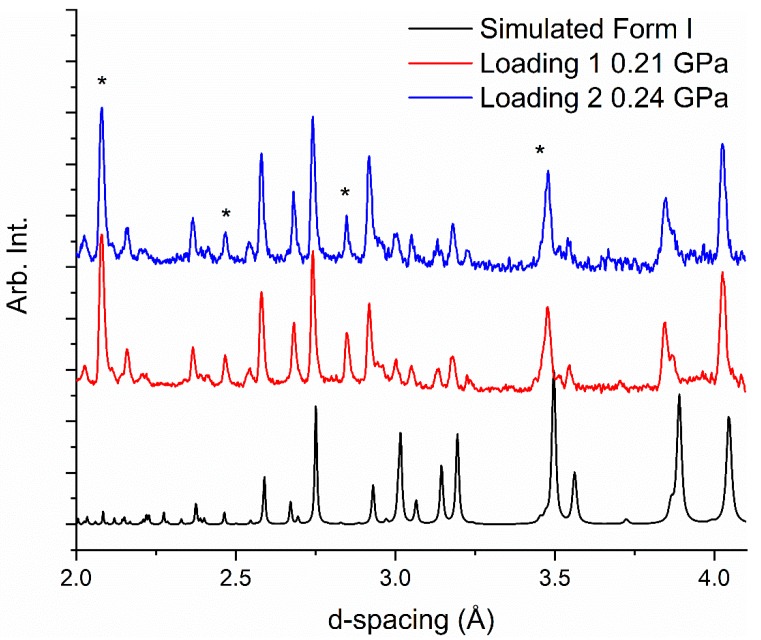
Neutron diffraction patterns of caprolactam-*d*_10_ (in d-spacing) from samples recrystallized from ethanol-*d*_6_ (top) and ethyl acetate (middle) together with a simulated pattern from the X-ray data from Winkler and Dunitz (bottom) [24]. The asterisks indicate the presence of alumina and lead that are part of the Paris-Edinburgh cell set-up. There is a peak of alumina that overlaps with a sample peak at 3.5 Å.

We conducted neutron-powder diffraction studies using the Pearl Instrument at ISIS Neutron and Muon Source at the Rutherford Appleton Laboratories, UK [27]. We used two loadings to explore the effects of solvent of recrystallisation and pressure. The first loading used a sample recrystallized from ethyl acetate and the second loading used a sample recrystallized from deuterated ethanol; 1-butanol was not available. Prior to loading, we recrystallized the sample in a mortar and pestle whilst gently grinding the powder to ensure that there was no preferential orientation of the crystallites. For both loadings, the caprolactam-*d*_10_ powder was loaded into a cooled titanium-zirconium encapsulated gasket that was placed in one of the zirconia-toughened alumina anvils. A pellet of lead was also added to the gasket to evaluate the pressure. A 1:1 mixture of perdeuterated pentane and isopentane was used as the pressure-transmitting medium to ensure that the compression was quasi-hydrostatic. The gasket and anvil assembly was loaded into a Paris-Edinburgh V3 press and a load applied to ensure that the gasket was sealed. Both loadings at ~0.2 GPa are consistent with Form I of caprolactam (Figure 2). Rietveld analysis of the samples showed that the caprolactam was not fully deuterated with the amide hydrogen being protonated.

### 2.4. Loading 1—Neutron Diffraction Study of Caprolactam-d_10_ Recrystallized from Ethyl Acetate

**Figure 3:**
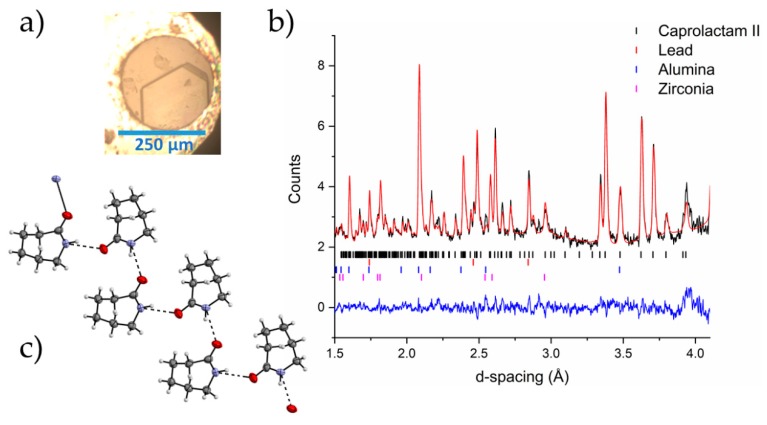
(**a**) A crystal of Form II of caprolactam grown from ethyl acetate from Form III crystals on decompression from 3.45 GPa to 0.55 GPa; (**b**) the Rietveld profile fit of Form II caprolactam-*d*_10_ to the neutron powder diffraction pattern collected at 0.943(11) GPa in 1:1 pentane:isopentane (crystals recrystallized from ethyl acetate); (**c**) the chain motif that is present in Form II of caprolactam.

During the single crystal experiment, using the lab diffractometer, we observed no changes to the crystal structure of caprolactam up to a pressure of 1.7 GPa. At 2.18 GPa, there was a loss of diffraction intensity that could be attributed to a reconstructive phase transition or a transformation to an amorphous solid. As we applied pressure to the sample in the neutron diffraction experiment, there was a change in the diffraction pattern at 0.685(11) GPa with complete conversion by 0.943(11) GPa. The new phase would not index to that of Form III that we had previously identified through single crystal diffraction of caprolactam (recrystallized from 1-butanol). Attempts to index and solve the new structure of the high-pressure phase, herein designated as Form II, from the neutron powder patterns were unsuccessful. Phase transitions that occur at these lower pressures are amenable to be explored using the pressure-induced precipitation methods due to the fact that the freezing pressures of solvents tend to be a little higher. This enables solutions to be loaded into pressure vessels and their solute precipitated into new high-pressure solid forms [28,29,30]. To identify Form II, we loaded a saturated solution of caprolactam in ethyl acetate into a Diamond Anvil Cell (DAC). We were not able to nucleate through pressure alone but the sample precipitated when we heated it to ca. 323 K. Multiple crystals were formed that were of sufficient quality for X-ray diffraction. These were identified as Form III (3.45 GPa). The cell was decompressed to ~0.7 GPa (where we observed Form II) but the sample was still Form III. We assumed that Form II was stable at this pressure and left the cell for two days at 0.7 GPa to allow for the possibility of recrystallisation. During this period, a single crystal of Form II grew from solution (Figure 3) which we analyzed through single-crystal X-ray diffraction and identified it as a new phase (Form II). We applied the structure from our single crystal diffraction data to the neutron powder diffraction pattern of Form II and found that it was an excellent fit to the data. Form II shows a chain motif compared with the dimer in Form I. 

The structure of Form II is dominated by the hydrogen bonded chains between the amide groups along the *b*-direction (Figure 3c; −27.7 kJ·mol^−1^; energies from single crystal at 0.7 GPa). The chains are stacked along the *c*-direction with neighbouring chains related by an inversion centre. The ½ unit cell offset between the neighbouring chains and the tilt of the molecules allows for the same H…O interaction that was observed in Form I where the axial hydrogen atoms are in close proximity to the carbonyl oxygen (−18.7 kJ·mol^−1^). The central molecule interacts with nearest neighbour along the *a*-direction through a dispersive interaction where there is a little overlap between amide moieties, which makes it a reasonably strong interaction (−21.1 kJ·mol^−1^). These weaker and less directional dispersive interactions will be affected to a greater extent as the crystal structure compresses under higher load.

**Figure 4:**
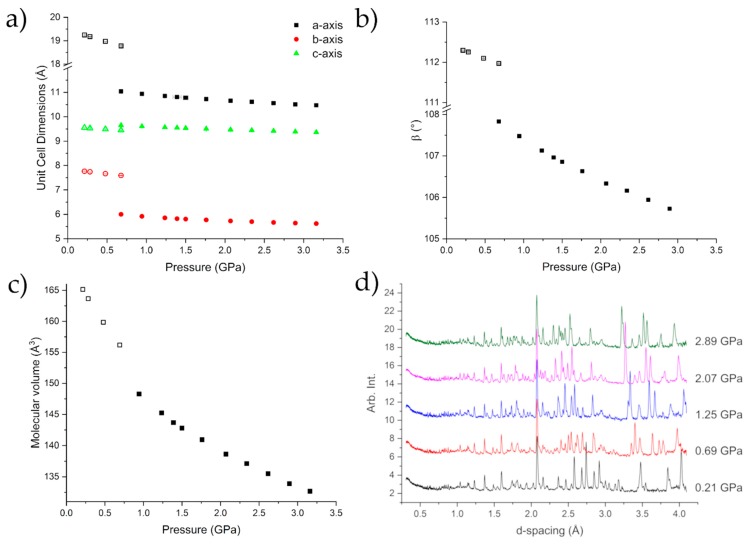
Compression of the unit cell dimensions (**a**,**b**) indicating the phase transition to Form II caprolactam-*d*_10_. There is a discontinuity in the molecular volume over the transition (**c**). The diffraction patterns of caprolactam as a function of pressure indicating the mixed phase 0.692(14) GPa (**d**). The error bars are smaller than the symbols.

Figure 4 shows the compression of Form I up to 0.678(8) GPa, at which point caprolactam undergoes a phase transition to Form II. A steady compression of all three axes is shown either side of the transition, with clear discontinuities in each axis, as well as the beta angle, as the sample undergoes the phase transition. Form II can be fit with a 3rd Order Murnaghan Equation of State with parameters V_0_ = 660(9) Å^3^, K = 5.1(16) GPa, K’ = 11(3) [31]. These values are commensurate with respect to values obtained for similar molecular systems e.g., glycolide (6.6 GPa) [20] and aniline (5.39 GPa) [32]. The errors associated with the parameters are a little higher due to the lack of data at lower pressures. This can be an issue with fitting the Equation of State to high pressure phases but in this case the Equation of State fits very well (Figure S1; Table S2).

Beyond the transition point, further compression to 3.13 GPa showed no significant change in the observed diffraction pattern (Figure 4d). We do observe that Form III, which we observed using single-crystal diffraction, grows in to a small extent and contributes to the peaks at approximately 3.6 & 4 Å. This growth begins at 2.0 GPa but does not show any increase as the pressure was applied. The time for the diffraction data from 2–3.13 GPa to be collected was ~20 h so there was not a long time for the nucleation of Form III. 

**Figure 5:**
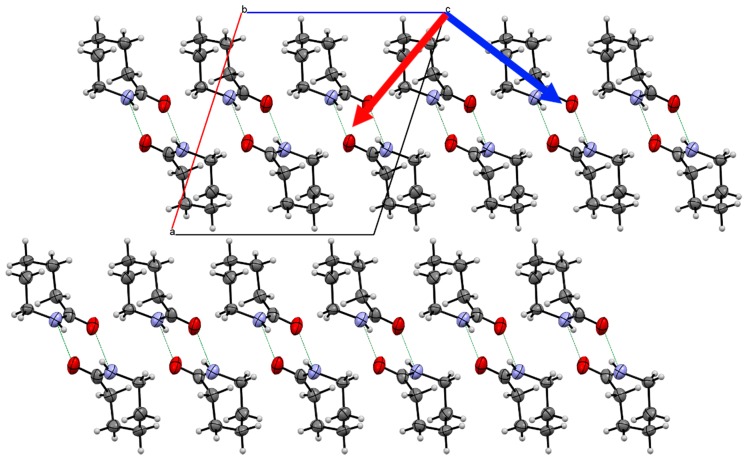
Crystal structure of Form II caprolactam viewed down the *b*-axis indicating two of the principal components of strain (Red arrow 19.1(2) TPa^−1^; Blue arrow 4.58(11) TPa^−1^). The third component is along the *b*-axis. The blue arrow indicates the direction of least compression. Whilst the molecules of one chain are positioned between the molecules of the neighbouring chain, the orientation of the molecules does not allow an increase in the degree of interlock.

In terms of the changes to structure with respect to pressure, these are more easily explained by the principal axis of strain. The strain tensor provides three directions of greatest compression in the crystal structure; one of which is along the [0 1 0] direction due to the symmetry of this polymorphic form. We observe that two of these axes are equally compressible [0 1 0] and the approximate [−2 0 1] direction but the third is significantly more rigid (18.8(4), 19.1(2) and 4.58(11) TPa^−1^) [33]. Compression along the [−2 0 1] direction pushes the hydrogen-bonded chains together where the soft van der Waals interactions are relatively easy to compress (Figure 5). The molecules are hydrogen bonded along the [0 1 0] direction; hence, one might assume that this would be rather rigid, however this is not the case. The position of the molecules in the chain allow them to be compressed towards each other like a spring. The lack of any other hydrogen-bonding groups on the alkyl group allows the molecule to move easily; hence, the hydrogen-bonded chain is able to act like a spring. The least compressible direction is along the approximate [1 0 2] direction. This direction is perpendicular to the chains and forces the chains together. There is not enough space for the chains to interlock further due to the orientation of the molecules. In addition, the molecules, in the same chain, are already in close proximity to one another due to the hydrogen-bonding interaction; hence any further compression is restricted.

Rapid decompression to 0.63(4) GPa shows large changes in the diffraction pattern (Figure S2). At this pressure, there is a mixed phase of both Forms I and II. The solid was maintained for only 20 min at this pressure, so there was not enough time for true equilibration to be reached. However, this pressure is similar to the pressure where the single crystal of Form II was formed and helps to define the lower boundary at which Form II is stable. We fully released the load on the sample to observe whether Form II could be recovered. After a short time period (10 min), it was clear that Form I was present. It should be noted that the data point at which the pressure refined to 0.24(3) GPa was in fact collected after the complete removal of the mechanical load. The fact that the pressure refined to 0.24(3) GPa can be attributed in part to the speed at which the load was removed, and in part the small quantity of data collected at this pressure.

### 2.5. Loading 2—Neutron Diffraction Study of Caprolactam Recrystallized from Ethanol

From the single-crystal experiment, we noted that the phase behaviour changed depending on the solvent of recrystallisation. To explore this further, we recrystallized caprolactam-*d*_10_ from deuterated ethanol; deuterated butanol was not available. On compression of this sample, we were able to observe Form I at two pressure points (0.235(13) GPa & 0.436(12) GPa) before a phase transition to Form III occurs at 0.94(4) GPa. At this pressure, we are able to partially fit Form II indicating that our initial hypothesis as to the role of the solvent was incorrect and that Form II has a narrow pressure range in which it is stable. Form II is still present at 1.54 GPa, but after this pressure was no longer observed. 

Form III possesses the same hydrogen-bond chain motif as in Form II (−35.6 kJ·mol^−1^; energies from single crystal at 0.7 GPa). Neighbouring chains along the *c*-direction interlock forming a layer of molecules in the *bc*-plane. The CH…O interaction that was observed in Form I and II is not present in this structure; instead, there is a greater face-to-face contact between the carbonyl groups of molecules in neighbouring chains that is equally as energetic as the interactions between hydrogen-bonded molecules (−37.0 kJ·mol^−1^). The layer is repeated along the *a*-axis direction forming a close-packed structure with the main interaction through the alkyl group of caprolactam (−6.8 to −8.5 kJ·mol^−1^). From inspection of the crystal structure, we would anticipate that the compression would be greatest between these layers.

**Figure 6:**
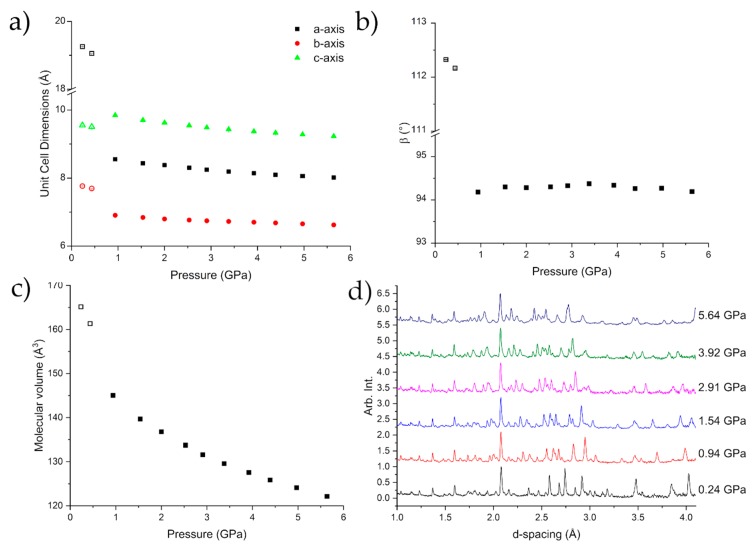
Compression of the unit cell dimensions (**a**,**b**,**d**) indicating the phase transition to Form III caprolactam. There is a discontinuity in the molecular volume over the transition (**c**). The error bars are smaller than the symbols.

Form III compresses monotonically until the highest pressure is achieved (5.64(2) GPa). Form III can be fit with a 3rd Order Murnaghan Equation of State with parameters V_0_ = 645(12) Å^3^, K = 4.7(19) GPa, K’ = 14(4) (Figure 6, Figure S3) [31]. The compressibility of Form III is similar to that of Form II whilst predicted to be a denser phase at ambient pressure. On decompression to ambient pressure Form III is present to 1.44(4) GPa but at the next pressure (0.53(4) GPa) Form II is present as the major phase; there are small misfits that can be assigned to Form III. Complete release of pressure shows the conversion to Form I (Figure S4).

**Figure 7:**
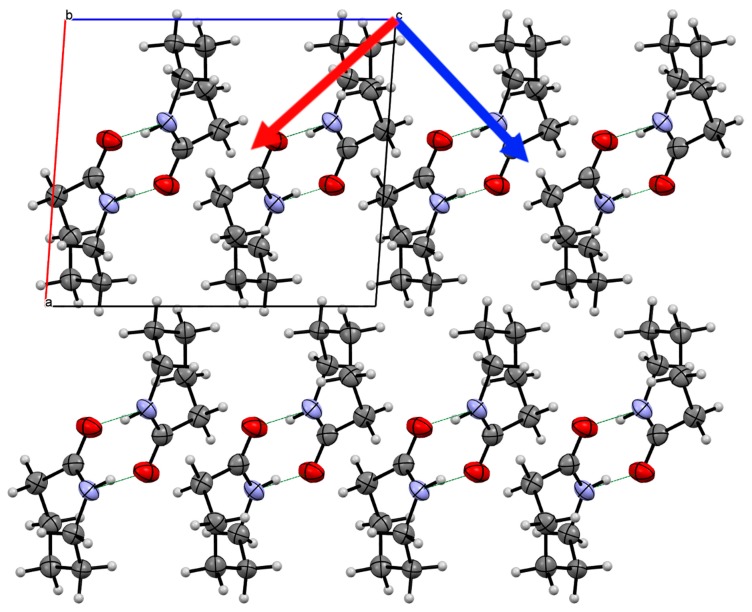
Crystal structure of Form III caprolactam viewed down the *b*-axis indicating the two of the principal components of strain (Red arrow 11.2(2) TPa^−1^; Blue arrow 10.3(3) TPa^−1^). The third component is along the *b*-axis. The two directions indicated are the most compressible in the structure. The packing of the chains is much greater than in Form II and the chains interlock to a greater extent. This allows the compression of the chains together, which reduces the compression of the hydrogen bonded chain (along the *b*-axis).

In Form III, we also observe that the compressibility is similar in two directions reflecting the simple hydrogen bonding pattern and the relative softness of the van der Waals interactions present. The approximate directions [−1 0 1] and [1 0 1] show the greatest compressibility with the [0 1 0] being the least compressible (11.2(2), 10.3(3) and 7.2(5) TPa^−1^) [33] (Figure 7). The main directions of compression are enabled by the soft van der Waals interactions that are made between the alkyl tail groups. The interlocking packing of the chains allows the chains to be compressed closer to each other without any direct contact between molecules. The least compressible direction is along the hydrogen bond direction. This is contrary to Form II but our only explanation to this is that the chains in Form III are interlocked to a greater extent, which prevents the hydrogen bond chain from being compressed.

## 3. Discussion

### Crystal Structure Comparison

**Figure 8:**
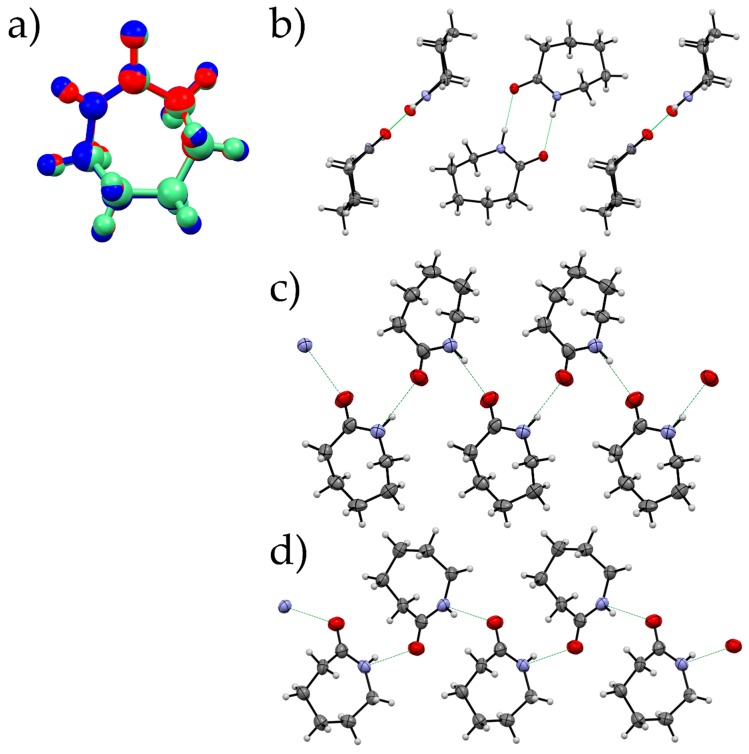
(**a**) An overlay of caprolactam molecules from three forms at 1.2 GPa created using Mercury [35] (Form I: Red, Form II: Blue, Form III: Green; RMS deviation between I and II 0.0310 and I and III 0.0131); hydrogen-bonding patterns for Form I (**b**), Form II (**c**), Form III (**d**). Form II and III showing the catemeric pattern with different orientations of molecules with respect to the direction of the chain.

The molecules in all the three structures show a similar conformation at ~1.2 GPa using the model from the Single crystal data for Forms I and III and the geometry optimised structure of Form II. However, the way in which they pack varies significantly between the three forms. The amide dimer arrangement, a prominent feature of Form I, is absent in both Forms II and III (Figure 8). Instead, Forms II and III consists of catemeric chains of the amide group, similar to those of carbamazepine form V [34]. The orientation of the molecules with respect to the hydrogen-bonded chains are different and can be measured through the N…O-C-N torsion angle (Form II, −106.35°; Form III, 105.34°). The change in the orientation of the molecules has an effect on the way in which the hydrogen-bonded chains interact with a substantial reorientation required to transform between Form II and Form III. This correlates with our observation that Form II can be retained at pressures beyond its region of stability. The orientation of the molecules also has an impact on the energy between hydrogen-bonded pairs where the Form III chain is ~7 kJ·mol^−1^ more favourable than in Form II (−38.1 kJ·mol^−1^ cf. −31.3 kJ·mol^−1^ Form II).

**Figure 9:**
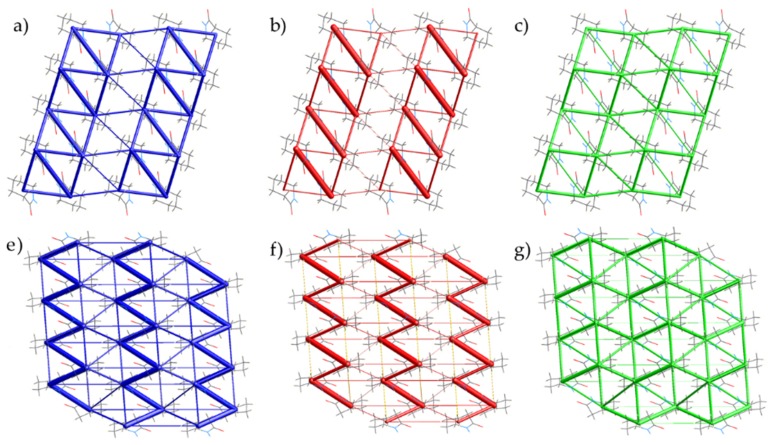
Energy frameworks for Forms II and III of caprolactam as calculated by CrystalExplorer [25]. Frameworks for the total energy (Form II, **a**; Form III, **e**), coulombic (Form II, **b**; Form III, **f**) and dispersive (Form II, **c**; Form III, **g**) contribution to the interactions. In each case, the view is along the hydrogen-bonded chain.

We calculated the intermolecular energies using CrystalExplorer [25] which gives more detail about the interactions in the solid. The calculations were performed using the 1.235(8) GPa and 0.94(4) GPa crystal structures of Form II and III, respectively, that have been subject to geometry optimization. In each of the forms, the interactions through the hydrogen bonded chain is the strongest interaction; however, there is a distinct difference in the energy between molecules of neighbouring chains. The change is associated with the interaction involving the carbonyl moieties of the amide groups. In Figure 9f, the interaction through the carbonyl groups is denoted by the second largest linking tube in Form III. In terms of total energy of interactions for Form III, there is little difference between the hydrogen-bonding interaction and the carbonyl-interaction (−38.1 vs. −38.6 kJ·mol^−1^); the spatial arrangement in Form II does not enable the carbonyl interaction. The importance of carbonyl-carbonyl interactions has been highlighted before by Allen and co-workers who used the Cambridge Structural Database to identify optimal geometries of the inter-carbonyl interaction and used calculations to show their comparative strength against hydrogen-bonding interactions [22,36]. Further work at high pressure has demonstrated its importance in amide complexes [37]. 

Whilst the carbonyl stacking helps to form stronger interaction between hydrogen bonding chains along the *c*-directions in both Forms, the interaction in the *a*-direction is much weaker due to the largely van der Waals interactions of the ring systems (Form II −7.6 kJ·mol^−1^; Form III −11.9 & −7.5 kJ·mol^−1^). The void space in both of the polymorphs resides in the area between chains. We observe that in Form II there is three times the void volume than in Form III at 1.235(8) GPa and 0.94(4) GPa, respectively, indicating that is far less dense at higher pressure, hence providing evidence that Form III is the more stable form at pressure (Figure S5). Throughout the compression range, the molecular volume of Form III is smaller than Form II, which is consistent with the lower void volume that we observe for Form III (Figure S6). The relative energies of each of the interactions remain consistent with increases in attraction across all the nearest neighbours. There is some indication that there may be another phase starting to appear with an unassigned peak at 3.6 Å; however, this is a minor component to the phase. There is no indication of the polymerization occurring in caprolactam with the recovery of Form I on decompression.

## 4. Materials and Methods

### 4.1. Lab-Based Experiments

A solvent screen was set up in which caprolactam (Sigma-Aldrich, Gillingham, UK) was recrystallised via slow solvent evaporation from acetone, acetonitrile, ethyl acetate, ethanol, methanol, and 1-butanol. The crystals obtained were screened using Raman spectroscopy and those suitable for single-crystal analysis (SXRD) were also analysed using this technique. All Raman spectra showed the same vibrational modes, indicating that no novel polymorphs had been obtained by recrystallization from the different solvents. The only samples that produced crystals of sufficient quality for analysis via SXRD were the samples recrystallized from ethyl acetate and 1-butanol. Analysis of crystals obtained from each of these recrystallized samples showed the crystals to match the known form of caprolactam.

A single crystal of caprolactam, produced via slow solvent evaporation of ethyl acetate, was loaded into a Merrill-Bassett DAC (University of Strathclyde, Glasgow, UK based on drawings provided by University of Edinburgh, Edinburgh, UK) equipped with 600 μm culets and a 250 μm sample chamberdrilled in a tungsten foil (250 μm thick). Petroleum ether was used as the pressure-transmitting medium to maintain hydrostatic conditions. SXRD and Raman data were taken at pressures of 0.64 GPa and 1.26 GPa. SXRD data obtained at both pressure points showed the sample to be in the known crystal form, herein designated as Form I. A third data collection, at 2.2 GPa showed that the sample quickly lost its crystallinity and that the intense spots associated with single-crystal diffraction were no longer present.

Ambient pressure analysis of a crystal obtained via slow evaporation of 1-butanol showed the crystal structure to again match that of the known phase. This crystal was then loaded into a DAC and analysed at 1.20 GPa, with the data obtained showing a phase transition had occurred. Data collected at 1.70 GPa and 2.18 GPa showed this second polymorph to still be present. Each of these compression studies were performed over the course of a week.

### 4.2. X-ray Diffraction Experiments

X-ray diffraction intensities were collected on a Bruker Kappa APEX2 diffractometer (Bruker, Coventry, UK) equipped with an Incoatec IµS molybdenum source (0.71073Å). The data were reduced using APEX3 software package (APEX3 v2018.1-0, Bruker AXS, Madison, WI, USA) applying the dynamic masking procedure within the system. Absorption correction was applied using SADABS [38] as incorporated in APEX3 suite. The model was either taken from the CSD (CAPLAC [24]) or solved using ShelXT [39] in Olex2 refinement package [40]. The non-hydrogen atoms were refined anisotropically and the hydrogen atoms placed on the parent atoms and allowed to ride. RIGU restraints were applied for all the structures.

### 4.3. Neutron Diffraction Experiments

Caprolactam was recrystallized via slow solvent evaporation of ethyl acetate and ethanol-*d*_6_. Deuterated ethanol was used due to the unavailability of 1-butanol in its deuterated form. Since there is no labile proton that could exchange with the sample, hydrogenated ethyl acetate was used. Samples were gently ground and loaded into a Paris-Edinburgh press as per procedures outlined in Hutchison et al. [20] using a lead pellet as the pressure marker and drops of a 1:1 (*v*/*v*) mixture of pentane and iso-pentane as a PTM. The Paris-Edinburgh press was sealed to ensure no leakage of the PTM before the assembly was craned into the Pearl beamline. Neutron diffraction data was collected at regular pressure intervals to a maximum pressure of 3.25 GPa and 5.69 GPa for the samples recrystallized from ethyl acetate and ethanol-*d*_6_, respectively (Table S2). Data were also collected on decompression in each case. The disparity in maximum pressure was a result of the limited time and pressure step size. The load was increased in increments of 2–3 tonnes during the first experiment (with the sample recrystallized from ethyl acetate). The maximum load (39 tonnes) was achieved after approximately 48 h, before decompression to ambient conditions over approximately 4 h. After the second loading (with the sample recrystallized from ethanol), the load was increased in increments of 5 tonnes. The maximum load (60 tonnes) was achieved after approximately 28 h before subsequent decompression to ambient conditions over the course of approximately 2 h. The data were refined in TOPAS academic [41] using a rigid body approach. The rigid body was generated from CAPLAC [24] or from the solved structures of our single-crystal determinations at high pressure. The rigid body was allowed free movement in translation and rotation. After successful refinement, the structures were output and geometry optimized to provide internal bond lengths and angles for a further final refinement of the atomic coordinates. The bond lengths, angles and torsional angles and their errors in the .cif files were calculated through the CALC ALL routine in Platon [42].

### 4.4. Geometry Optimisation

Geometry optimizations were performed by periodic Density Functional Theory (DFT) using the DMOL^3^ code [43] as part of the Materials Studio 2018 package [44]. The DNP numerical basis set [43] was used in combination with the PBE [45] functional with the Tkatchenko-Scheffler correction [46] for dispersion. The unit cell dimensions were held fixed at the values obtained in Pawley or Rietveld refinements of the neutron powder diffraction data, and coordinates were allowed to optimize. Convergence was defined when the maximum changes in total energy, displacement and gradient were 10^−5^ Ha, 5 × 10^−3^ Å and 2 × 10^−3^ Ha Å^−1^, respectively. Brillouin zone integrations were performed by Monkhorst-Pack **k**-point sampling at intervals of 0.07 Å^−1^. 

### 4.5. Crystal Explorer

The geometry optimized structures were used to calculate the intermolecular energies using CrystalExplorer (University of Western Australia, Perth, Australia) [25]. A default radius of 3.8 Å was used to generate the cluster before using the CE-B3LYP/6-31G(d,p) energy model. 

## 5. Conclusions

Single-crystal and neutron diffraction studies show that caprolactam undergoes two phase transitions to Form II and Form III (both *P*2_1_/*c*). Through the combination of these techniques, we observed that Form II is stable over a narrow pressure range between approximately 0.5 GPa and 0.9 GPa, but kinetics plays a role in its observation. Once formed, Form II can be compressed beyond the phase boundary due to the large reconstructive transition that is required to convert to the high-pressure Form III phase. Form II can be bypassed by compression beyond the phase boundary so that the reconstructive phase transition from Form I to III can take place. In our case, the compression from 0.44 GPa to 0.94 GPa was over a period of five minutes; hence, Form II did not have time to nucleate. Form III demonstrates that the carbonyl-carbonyl interaction is extremely important and can contribute substantially to the overall energy of the system. The reaction of the hydrogen-bonded chains in Form II and III to high pressure are very different due to the packing of the chains with respect to each other. Form II shows little intercalating of the chains; hence, on compression the hydrogen bonded chain can compress easily. In Form III, the molecules in the chain are much more intercalated; hence, on compression the hydrogen-bonded chain cannot compress and the crystal structure shows a lower compressibility in this direction. In either of these studies, no polymerization was observed in this system.

## Supplementary Materials

The following are available online, Figure S1: The 3rd Order Birch-Murnaghan Equation of State for Form II of caprolactam. Data points from the Pawley and Rietveld fits of the neutron diffraction data collected on the crystals obtained from ethyl acetate and with perdeuterated pentane/isopentane as the pressure-transmitting medium, Figure S2: The decompression of Form II of caprolactam. At 0.63 GPa is a mixed phase of Form I and II and the transition to Form I is complete by 0.24 GPa. This was the lowest pressure achieved on decompression with the load taken off the sample. The sample was not left for any length of time so the pressure will not have had time to equilibrate, Figure S3: The 3rd Order Birch-Murnaghan Equation of State for Form III of caprolactam. Data points from the Pawley and Rietveld fits of the neutron diffraction data collected on the crystals obtained from ethanol-d6 and with perdeuterated pentane/isopentane as the pressure-transmitting medium, Figure S4: (a) The Pawley fit of the data at 2 tonnes on decompression (0.53 GPa) using the cell parameters of Form III of caprolactam; (b) the diffraction patterns of caprolactam on decompression from 5.69 GPa, Figure S5: Void space using 0.5 Å probe radius and 0.2 grid spacing for (a) Form II at 1.2 GPa and (b) Form III at 0.94 GPa. The percentage of void space in Form II was 4% of unit cell volume compared with 1.5% for Form III, Figure S6: The molecular volume of each form as a function of pressure. The two loadings are color coded black (Loading 1; crystal from ethyl acetate) and red (Loading 2; crystal from ethanol-*d*_6_). Hollow symbols represent Form I and solid symbols represent Forms II and III. Form III is consistently denser over the entire compression range, Figure S7: Rietveld fit of the 5.4 GPa data to Form III. The peak at 4.1 Å is due to the sample. The model used was taken from the geometry optimized structure and the orientation allowed to refine whilst keeping conformation of the molecule fixed, Table S1: Crystallographic details for the X-ray determined structures, Table S2: The unit cell parameters of caprolactam-*d*_10_ on compression derived from neutron powder diffraction data collected on the Pearl instrument.

## References

[1] Murthy N.S. (2006). Hydrogen bonding, mobility, and structural transitions in aliphatic polyamides. J. Polym. Sci. Part B Polym. Phys..

[2] Rahman M.A., Renna L.A., Venkataraman D., Desbois P., Lesser A.J. (2018). High crystalline, porous polyamide 6 by anionic polymerization. Polymer.

[3] Fernandes M.A., Levendis D.C. (2016). Photodimerisation of the α′-polymorph of ortho-ethoxy-trans-cinnamic acid occurs via a two-stage mechanism at 343 K yielding 100% α-truxillic acid. CrystEngComm.

[4] Sinnwell M.A., Blad J.N., Thomas L.R., MacGillivray L.R. (2018). Structural flexibility of halogen bonds showed in a single-crystal-to-single-crystal [2 + 2] photodimerization. IUCrJ.

[5] Murli C., Song Y. (2010). Pressure-Induced Polymerization of Acrylic Acid: A Raman Spectroscopic Study. J. Phys. Chem. B.

[6] Murli C., Mishra A.K., Thomas S., Sharma S.M. (2012). Ring-Opening Polymerization in Carnosine under Pressure. J. Phys. Chem. B.

[7] Chelazzi D., Ceppatelli M., Santoro M., Bini R., Schettino V. (2005). Pressure-Induced Polymerization in Solid Ethylene. J. Phys. Chem. B.

[8] Ciabini L., Gorelli F.A., Santoro M., Bini R., Schettino V., Mezouar M. (2005). High-pressure and high-temperature equation of state and phase diagram of solid benzene. Phys. Rev. B.

[9] Santoro M., Ciabini L., Bini R., Schettino V. (2003). High-Pressure Polymerization of Phenylacetylene and of the Benzene and Acetylene Moieties. J. Raman Spectrosc..

[10] Ciabini L., Santoro M., Bini R., Schettino V. (2002). High Pressure Reactivity of Solid Benzene Probed by Infrared Spectroscopy. J. Chem. Phys..

[11] Guan J., Daljeet R., Kieran A., Song Y. (2018). Pressure-induced amorphization and reactivity of solid dimethyl acetylene probed by in situ FTIR and Raman spectroscopy. J. Phys. Condens. Matter.

[12] Sun J., Dong X., Wang Y., Li K., Zheng H., Wang L., Cody G.D., Tulk C.A., Molaison J.J., Lin X. (2017). Pressure-Induced Polymerization of Acetylene: Structure-Directed Stereoselectivity and a Possible Route to Graphane. Angew. Chem. Int. Ed..

[13] Delori A., Hutchison I.B., Bull C.L., Funnell N.P., Urquhart A.J., Oswald I.D.H. (2018). Reaction of Acetylenedicarboxylic Acid Made Easy: High-Pressure Route for Polymerization. Cryst. Growth Des..

[14] Bini R., Ceppatelli M., Citroni M., Schettino V. (2012). From Simple to Complex and Backwards. Chemical Reactions under Very High Pressure. Chem. Phys..

[15] Dziubek K.F., Ende M., Scelta D., Bini R., Mezouar M., Garbarino G., Miletich R. (2018). Crystalline polymeric carbon dioxide stable at megabar pressures. Nat. Commun..

[16] Wilhelm C., Boyd S.A., Chawda S., Fowler F.W., Goroff N.S., Halada G.P., Grey C.P., Lauher J.W., Luo L., Martin C.D. (2008). Pressure-Induced Polymerization of Diiodobutadiyne in Assembled Cocrystals. J. Am. Chem. Soc..

[17] Jin H.J., Plonka A.M., Parise J.B., Goroff N.S. (2013). Pressure Induced Topochemical Polymerization of Diiodobutadiyne: A Single-Crystal-to-Single-Crystal Transformation. CrystEngComm.

[18] Langer R., Vacanti J.P. (1993). Tissue engineering. Science.

[19] Hutchison I.B., Delori A., Wang X., Kamenev K.V., Urquhart A.J., Oswald I.D.H. (2015). Polymorphism of a polymer precursor: Metastable glycolide polymorph recovered via large scale high-pressure experiments. CrystEngComm.

[20] Hutchison I.B., Bull C.L., Marshall W.G., Parsons S., Urquhart A.J., Oswald I.D.H. (2017). Compression of glycolide-h4to 6GPa. Acta Crystallogr. Sect. B Struct. Sci. Cryst. Eng. Mater..

[21] Oya K.P., Myasnikova R.M. (1974). Crystals of binary molecular compounds formed by hydrogen bonds—III. Crystal structure of e{open}-caprolactam. J. Struct. Chem..

[22] Groom C.R., Bruno I.J., Lightfoot M.P., Ward S.C. (2016). The Cambridge Structural Database. Acta Crystallogr. Sect. B Struct. Sci. Cryst. Eng. Mater..

[23] Assarsson P. (1962). Space group of ɛ-caprolactam (2-oxohexamethylenimine). J. Polym. Sci..

[24] Winkler F.K., Dunitz J.D. (1975). Medium-Ring Compounds. XIX. Caprolactam: Structure Refinement. Acta Crystallogr..

[25] Thomas S.P., Spackman P.R., Jayatilaka D., Spackman M.A. (2018). Accurate Lattice Energies for Molecular Crystals from Experimental Crystal Structures. J. Chem. Theory Comput..

[26] Garbuzova I.A., Lokshin B.V. (2004). Hydrogen bonding in ε-caprolactam dimer: A quantum-chemical study. Russ. Chem. Bull..

[27] Bull C.L., Funnell N.P., Tucker M.G., Hull S., Francis D.J., Marshall W.G. (2016). PEARL: The high pressure neutron powder diffractometer at ISIS. High Press. Res..

[28] Fabbiani F.P.A., Allan D.R., Dawson A., David W.I.F., McGregor P.A., Oswald I.D.H., Parsons S., Pulham C.R. (2003). Pressure-induced formation of a solvate of paracetamol. Chem. Commun..

[29] Oswald I.D.H., Pulham C.R. (2008). Co-crystallisation at high pressure—An additional tool for the preparation and study of co-crystals. CrystEngComm.

[30] Fabbiani F.P.A., Buth G., Levendis D.C., Cruz-Cabeza A.J. (2014). Pharmaceutical hydrates under ambient conditions from high-pressure seeds: A case study of GABA monohydrate. Chem. Commun..

[31] Gonzalez-Platas J., Alvaro M., Nestola F., Angel R. (2016). EosFit7-GUI: A new graphical user interface for equation of state calculations, analyses and teaching. J. Appl. Crystallogr..

[32] Funnell N.P., Dawson A., Marshall W.G., Parsons S. (2013). Destabilisation of hydrogen bonding and the phase stability of aniline at high pressure. CrystEngComm.

[33] Cliffe M.J., Goodwin A.L. (2012). PASCal: A principal axis strain calculator for thermal expansion and compressibility determination. J. Appl. Crystallogr..

[34] Arlin J.-B., Price L.S., Price S.L., Florence A.J., Price S.L., Dunitz J.D., Scheraga H.A., Byrn S.R., Pfeiffer R.R., Stephenson G. (2011). A strategy for producing predicted polymorphs: Catemeric carbamazepine form V. Chem. Commun..

[35] Macrae C.F., Bruno I.J., Chisholm J.A., Edgington P.R., McCabe P., Pidcock E., Rodriguez-Monge L., Taylor R., van de Streek J., Wood P.A. (2008). Mercury CSD 2.0—New features for the visualization and investigation of crystal structures. J. Appl. Crystallogr..

[36] Allen F.H., Baalham C.A., Lommerse J.P.M., Raithby P.R. (1998). Carbonyl–Carbonyl Interactions can be Competitive with Hydrogen Bonds. Acta Crystallogr. Sect. B Struct. Sci..

[37] Wood P.A., Haynes D.A., Lennie A.R., Samuel W.D., Parsons S., Pidcock E., Warren J.E. (2008). The Anisotropic Compression of the Crystal Structure of 3-Aza-bicyclo(3.3.1)nonane-2,4-dione to 7.1 GPa. Cryst. Growth Des..

[38] Sheldrick G.M. (2008). SADABS, Programs for Scaling and Absorption Correction of Area Detector Data.

[39] Sheldrick G.M. (2015). IUCr SHELXT—Integrated space-group and crystal-structure determination. Acta Crystallogr. Sect. A Found. Adv..

[40] Dolomanov O.V., Bourhis L.J., Gildea R.J., Howard J.A.K., Puschmann H. (2009). OLEX2: A complete structure solution, refinement and analysis program. J. Appl. Crystallogr..

[41] Coelho A. (2012). TOPAS—Academic: General Profile and Structure Analysis Software for Powder Diffraction Data.

[42] Spek A.L. (2009). Structure validation in chemical crystallography. Acta Crystallogr. Sect. D Biol. Crystallogr..

[43] Delley B. (1990). An All-Electron Numerical-Method for Solving the Local Density Functional for Polyatomic-Molecules. J. Chem. Phys..

[44] BIOVIA (2018). D.S. Materials Studio 2018.

[45] Perdew J.P., Chevary J.A., Vosko S.H., Jackson K.A., Pederson M.R., Singh D.J., Fiolhais C. (1992). Atoms, Molecules, Soilds, and Surfaces—Applications of the Generalized Gradient Approximation for Exchange and Correlation. Phys. Rev. B.

[46] Tkatchenko A., Scheffler M. (2009). Accurate Molecular Van Der Waals Interactions from Ground-State Electron Density and Free-Atom Reference Data. Phys. Rev. Lett..

[47] Oswald I.D.H., Connor L.E., Marshall W.G., Hutchison I.B., Bull C.L., Urquhart A.J. Investigation of Caprolactam-D11 at High Pressure. https://data.isis.stfc.ac.uk/doi/investigation/73945863.

