# Supplementary material for: Pressure-Induced Polymorphism of Caprolactam: A Neutron Diffraction Study
Source: Molecules. 2019 Jun 10;24(11):2174. doi: 10.3390/molecules24112174 (PMC6600225; doi:10.3390/molecules24112174)
Supplement: Supplementary file 1 [file molecules-24-02174-s001.pdf]

molecules

MDPI

*Supplementary Materials*

# Pressure-induced Polymorphism of Caprolactam: A Neutron Diffraction Study

**Ian B. Hutchison [1], Craig L. Bull [2], William G. Marshall [2,‡], Andrew J. Urquhart [3] and Iain D.H. Oswald [1,*]**

[1] Strathclyde Institute of Pharmacy & Biomedical Sciences (SIPBS), University of Strathclyde, 161 Cathedral Street, Glasgow G4 0RE, UK; ian.b.hutchison@gmail.com
[2] ISIS Neutron and Muon Source, Science and Technology Facilities Council, Rutherford Appleton Laboratory, Harwell, Didcot OX11 0QX, UK; craig.bull@stfc.ac.uk (C.L.B.);
[3] Department of Health Technology, Technical University of Denmark, Produktionstorvet, 2800 Kgs. Lyngby, Denmark; anur@dtu.dk
* Correspondence: iain.oswald@strath.ac.uk; Tel.: +44-0141-548-2157
‡ Deceased on 5 November 2015

**Table S1.** Crystallographic details for the X-ray determined structures. For all structures: $C_6H_{11}NO$, $M_r$ = 113.16. Experiments were carried out on a Bruker Kappa Apex2 diffractometer with Mo *Kα* radiation. Refinement was on 73 parameters. H-atom parameters were constrained.

| | EA1 | EA2 | EA3 | But1 |
|---|---|---|---|---|
| Crystal data | | | | |
| Crystal system, space group | Monoclinic, *C*2/*c* | Monoclinic, *C*2/*c* | Monoclinic, *C*2/*c* | Monoclinic, $P2_1/c$ |
| Temperature (K) | 123 | 300 | 300 | 300 |
| Pressure (GPa) | ambient | 0.64 | 1.63 | 1.20 |
| *a*, *b*, *c* (Å) | 18.8703 (18), 7.6517 (7), 9.5444 (9) | 18.8380 (18), 7.6205 (4), 9.4652 (5) | 18.3060 (16), 7.4101 (3), 9.3417 (4) | 8.480 (1), 6.8718 (5), 9.7502 (19) |
| β (°) | 111.538 (4) | 112.023 (4) | 111.924 (4) | 94.219 (12) |
| *V* (Å$^3$) | 1281.9 (2) | 1259.63 (16) | 1175.55 (13) | 566.63 (14) |
| *Z* | 8 | 8 | 8 | 4 |
| μ (mm$^{-1}$) | 0.08 | 0.08 | 0.09 | 0.09 |
| Crystal size (mm) | 0.2 × 0.15 × 0.1 | 0.2 × 0.1 × 0.05 | 0.2 × 0.1 × 0.05 | 0.2 × 0.1 × 0.05 |
| | | | | |
| Data collection | | | | |
| Diffractometer | Bruker *SMART APEX2* area detector | Bruker *SMART APEX2* area detector | Bruker *SMART APEX2* area detector | Bruker *SMART APEX2* area detector |
| Absorption correction | Multi-scan *SADABS2016*/2 (Bruker,2016/2) was used for absorption correction. wR2(int) was 0.0572 before and 0.0336 after correction. The Ratio of minimum to | Multi-scan *SADABS2016*/2 (Bruker,2016/2) was used for absorption correction. wR2(int) was 0.0569 before and 0.0376 after correction. The Ratio of minimum to | Multi-scan *SADABS2016*/2 (Bruker,2016/2) was used for absorption correction. wR2(int) was 0.0551 before and 0.0402 after correction. The Ratio of minimum to | Multi-scan *SADABS2016*/2 (Bruker,2016/2) was used for absorption correction. wR2(int) was 0.0686 before and 0.0458 after correction. The Ratio of minimum to |

| | | | | |
|---|---|---|---|---|
| | maximum transmission is 0.9423. The λ/2 correction factor is Not present. | maximum transmission is 0.9176. The λ/2 correction factor is Not present. | maximum transmission is 0.9165. The λ/2 correction factor is Not present. | maximum transmission is 0.9000. The λ/2 correction factor is Not present. |
| $T_{min}$, $T_{max}$ | 0.702, 0.745 | 0.684, 0.745 | 0.683, 0.745 | 0.670, 0.745 |
| No. of measured, independent and observed [$I > 2\sigma(I)$] reflections | 5777, 1317, 1213 | 2843, 475, 405 | 2630, 444, 393 | 2570, 306, 256 |
| $R_{int}$ | 0.020 | 0.025 | 0.024 | 0.038 |
| $\theta_{max}$ (°) | 26.4 | 23.2 | 23.3 | 23.3 |
| $(\sin \theta/\lambda)_{max}$ (Å$^{-1}$) | 0.625 | 0.555 | 0.556 | 0.555 |
| | | | | |
| Refinement | | | | |
| $R[F^2 > 2\sigma(F^2)]$, $wR(F^2)$, $S$ | 0.033, 0.087, 1.11 | 0.029, 0.071, 1.11 | 0.030, 0.078, 1.03 | 0.030, 0.078, 1.10 |
| No. of reflections | 1317 | 475 | 444 | 306 |
| No. of restraints | 0 | 51 | 51 | 51 |
| Data completeness | 100% | 52% | 53% | 37% |
| $_{max}$, $_{min}$ (e Å$^{-3}$) | 0.25, -0.17 | 0.06, -0.06 | 0.09, -0.06 | 0.07, -0.07 |

| | But2 | But3 | Recryst1 | Recryst2 |
|---|---|---|---|---|
| Crystal data | | | | |
| Crystal system, space group | Monoclinic, $P2_1/c$ | Monoclinic, $P2_1/c$ | Monoclinic, $P2_1/c$ | Monoclinic, $P2_1/c$ |
| Temperature (K) | 300 | 300 | 300 | 296 |
| Pressure (GPa) | 1.70 | 2.18 | 0.7 | 0.7 |
| $a$, $b$, $c$ (Å) | 8.4244 (14), 6.8435 (6), 9.674 (2) | 8.3465 (13), 6.8128 (14), 9.5998 (15) | 8.651 (3), 6.948 (2), 9.950 (2) | 11.0794 (19), 6.0418 (3), 9.6662 (5) |
| β (°) | 94.322 (15) | 94.289 (13) | 94.03 (2) | 107.896 (9) |
| $V$ (Å$^3$) | 556.13 (16) | 544.35 (16) | 596.5 (3) | 615.74 (12) |
| $Z$ | 4 | 4 | 4 | 4 |
| μ (mm$^{-1}$) | 0.09 | 0.09 | 0.09 | 0.08 |
| Crystal size (mm) | 0.2 × 0.1 × 0.05 | 0.2 × 0.1 × 0.05 | 0.2 × 0.15 × 0.05 | 0.2 × 0.15 × 0.05 |
| | | | | |
| Data collection | | | | |
| Diffractometer | Bruker *SMART APEX2* area detector | Bruker *SMART APEX2* area detector | Bruker *APEX*-II CCD | Bruker *SMART APEX2* area detector |
| Absorption correction | Multi-scan *SADABS2016*/2 (Bruker,2016/2) was used for absorption correction. wR2(int) was | Multi-scan *SADABS2016*/2 (Bruker,2016/2) was used for absorption correction. wR2(int) was | Multi-scan *SADABS2016*/2 (Bruker,2016/2) was used for absorption correction. wR2(int) was | Multi-scan *SADABS2016*/2 (Bruker,2016/2) was used for absorption correction. wR2(int) was |

| | | | | |
|---|---|---|---|---|
| | 0.1202 before and 0.0530 after correction. The Ratio of minimum to maximum transmission is 0.8670. The λ/2 correction factor is Not present. | 0.1450 before and 0.0577 after correction. The Ratio of minimum to maximum transmission is 0.6867. The λ/2 correction factor is Not present. | 0.0905 before and 0.0355 after correction. The Ratio of minimum to maximum transmission is 0.9144. The λ/2 correction factor is Not present. | 0.0571 before and 0.0385 after correction. The Ratio of minimum to maximum transmission is 0.9199. The λ/2 correction factor is Not present. |
| $T_{min}$, $T_{max}$ | 0.646, 0.745 | 0.511, 0.745 | 0.681, 0.745 | 0.685, 0.745 |
| No. of measured, independent and observed [$I > 2\sigma(I)$] reflections | 2373, 284, 220 | 2137, 401, 302 | 2691, 451, 352 | 2691, 319, 293 |
| $R_{int}$ | 0.047 | 0.055 | 0.037 | 0.024 |
| $\theta_{max}$ (°) | 23.2 | 23.3 | 23.3 | 23.3 |
| $(\sin \theta/\lambda)_{max}$ (Å$^{-1}$) | 0.554 | 0.557 | 0.556 | 0.556 |
| | | | | |
| Refinement | | | | |
| $R[F^2 > 2\sigma(F^2)]$, $wR(F^2)$, $S$ | 0.039, 0.106, 1.23 | 0.045, 0.142, 0.90 | 0.039, 0.108, 1.09 | 0.027, 0.072, 1.17 |
| No. of reflections | 284 | 401 | 451 | 319 |
| No. of restraints | 51 | 51 | 51 | 0 |
| Data completeness | 36% | 51% | 53% | 36% |
| $\Delta\rangle_{max}$, $\Delta\rangle_{min}$ (e Å$^{-3}$) | 0.10, -0.09 | 0.18, −0.15 | 0.13, −0.12 | 0.05, −0.05 |

**Table S2.** The unit cell parameters of caprolactam-*d₁₀* on compression derived from neutron powder diffraction data collected on the Pearl instrument. Two loading were performed using perdeuterated pentane and isopentane as the pressure transmitting medium.

| Pressure (GPa) | Polymorph | a-axis (Å) | b-axis (Å) | c-axis (Å) | β (°) | Volume (Å$^3$) | $r_{wp}$ |
|---|---|---|---|---|---|---|---|
| | Loading 1 – crystals from ethyl acetate | | | | | | |
| 0.213(9) | I | 19.2487(16) | 7.7647(6) | 9.5543(8) | 112.299(5) | 1321.2(2) | 2.965 |
| 0.283(7) | I | 19.1743(12) | 7.7397(9) | 9.5317(7) | 112.255(4) | 1309.2(2) | 1.736 |
| 0.479(9) | I | 18.9732(13) | 7.6630(11) | 9.4928(8) | 112.100(5) | 1278.8(2) | 1.891 |
| 0.678(8) | I | 18.780(3) | 7.5918(14) | 9.4522(17) | 111.970(12) | 1249.8(4) | 2.686 |
| 0.678(8) | II | 11.0391(9) | 5.9986(7) | 9.6526(11) | 107.830(9) | 608.48(12) | 2.686 |
| 0.943(11) | II | 10.9381(6) | 5.9179(5) | 9.6067(7) | 107.479(6) | 593.13(8) | 3.065 |
| 1.235(8) | II | 10.8506(5) | 5.8539(4) | 9.5704(6) | 107.129(5) | 580.93(6) | 2.559 |
| 1.39(5) | II | 10.8082(5) | 5.8201(6) | 9.5519(8) | 106.963(5) | 574.72(8) | 2.278 |
| 1.50(3) | II | 10.7801(5) | 5.8047(5) | 9.5371(7) | 106.858(5) | 571.14(7) | 2.171 |
| 1.761(14) | II | 10.7242(6) | 5.7704(5) | 9.5068(8) | 106.630(7) | 563.70(8) | 3.722 |
| 2.070(10) | II | 10.6537(5) | 5.7272(4) | 9.4719(7) | 106.334(7) | 554.61(7) | 1.325 |
| 2.070(10) | III | 8.446(2) | 6.781(4) | 9.560(3) | 94.52(3) | 545.9(3) | 1.325 |
| 2.340(11) | II | 10.6097(16) | 5.6984(11) | 9.4456(11) | 106.162(10) | 548.49(15) | 1.896 |
| 2.340(11) | III | 8.191(7) | 6.741(7) | 9.526(4) | 94.50(7) | 524.3(8) | 1.896 |
| 2.617(12) | II | 10.559(3) | 5.6664(13) | 9.4202(15) | 105.943(10) | 542.0(2) | 1.984 |
| 2.617(12) | III | 8.203(6) | 6.753(7) | 9.463(5) | 94.44(6) | 522.6(7) | 1.984 |
| 2.894(14) | II | 10.5085(6) | 5.6394(4) | 9.3892(5) | 105.726(5) | 535.59(6) | 1.843 |
| 2.894(14) | III | 8.175(3) | 6.740(2) | 9.469(3) | 94.44(3) | 520.2(3) | 1.843 |
| 3.16(3) | II | 10.4696(15) | 5.6185(12) | 9.3634(13) | 105.539(8) | 530.66(15) | 1.964 |
| 3.16(3) | III | 8.179(7) | 6.758(10) | 9.460(4) | 94.71(5) | 521.1(9) | 1.964 |
| 0.63(4) | I | 19.371(16) | 7.758(6) | 9.502(7) | 111.93(7) | 1324.6(19) | 4.793 |
| 0.63(4) | II | 11.013(6) | 6.045(8) | 9.593(7) | 107.735(17) | 608.2(10) | 4.793 |
| 0.24(3) | I | 19.261(6) | 7.767(3) | 9.553(3) | 112.344(18) | 1321.7(8) | 9.508 |
| | Loading 2 – crystals from ethanol-*d₆* | | | | | | |
| 0.235(13) | I | 19.2556(17) | 7.7647(9) | 9.5524(9) | 112.324(5) | 1321.2(2) | 2.599 |
| 0.436(12) | I | 19.0531(12) | 7.6934(9) | 9.5069(7) | 112.166(4) | 1290.6(2) | 1.976 |

| | | | | | | | |
|---|---|---|---|---|---|---|---|
| 0.94(4) | III | 8.5531(11) | 6.9059(7) | 9.8477(12) | 94.178(13) | 580.13(12) | 1.695 |
| 1.536(16) | III | 8.4376(9) | 6.8422(6) | 9.7049(7) | 94.298(10) | 558.70(9) | 1.535 |
| 2.001(15) | III | 8.3813(16) | 6.7978(8) | 9.6285(7) | 94.281(12) | 547.05(13) | 2.456 |
| 2.532(17) | III | 8.302(2) | 6.7669(10) | 9.5468(7) | 94.301(15) | 534.85(17) | 2.436 |
| 2.91(3) | III | 8.2477(8) | 6.7443(7) | 9.4882(9) | 94.326(11) | 526.28(9) | 3.693 |
| 3.381(18) | III | 8.1913(15) | 6.7262(9) | 9.4337(9) | 94.371(14) | 518.25(13) | 2.449 |
| 3.923(17) | III | 8.1435(15) | 6.7034(9) | 9.3730(13) | 94.338(14) | 510.20(14) | 2.251 |
| 4.39(2) | III | 8.097(2) | 6.6832(13) | 9.3284(15) | 94.26(2) | 503.40(19) | 3.107 |
| 4.97(5) | III | 8.0579(17) | 6.6547(11) | 9.2829(13) | 94.269(19) | 496.40(15) | 2.614 |
| 5.64(2) | III | 8.0125(7) | 6.6231(6) | 9.2301(9) | 94.189(10) | 488.51(8) | 2.989 |
| 4.99(3) | III | 8.0524(16) | 6.6547(10) | 9.2789(10) | 94.225(19) | 495.88(14) | 2.946 |
| 3.63(2) | III | 8.169(2) | 6.7201(14) | 9.391(2) | 94.37(2) | 514.1(2) | 3.295 |
| 1.46(3) | III | 8.442(5) | 6.857(2) | 9.7115(14) | 94.35(3) | 560.6(4) | 3.766 |
| 0.53(4) | II | 11.0490(8) | 6.0002(12) | 9.6644(16) | 107.787(8) | 610.09(17) | 3.066 |
| 0.21(5) | I | 19.243(4) | 7.767(2) | 9.544(2) | 112.279(15) | 1319.9(6) | 8.15 |

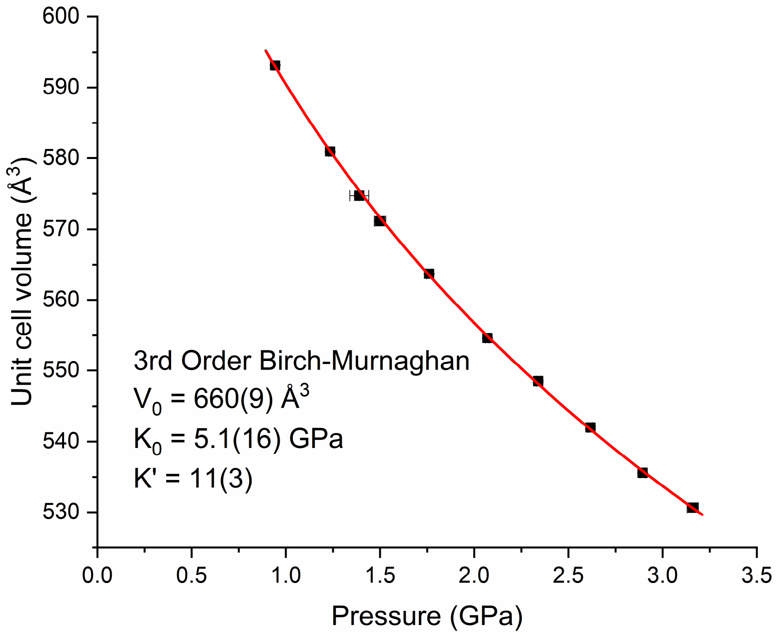


**Figure S1.** The 3rd Order Birch-Murnaghan Equation of State for Form II of caprolactam. Data points from the Pawley and Rietveld fits of the neutron diffraction data collected on the crystals obtained from ethyl acetate and with perdeuterated pentane/isopentane as the pressure-transmitting medium.

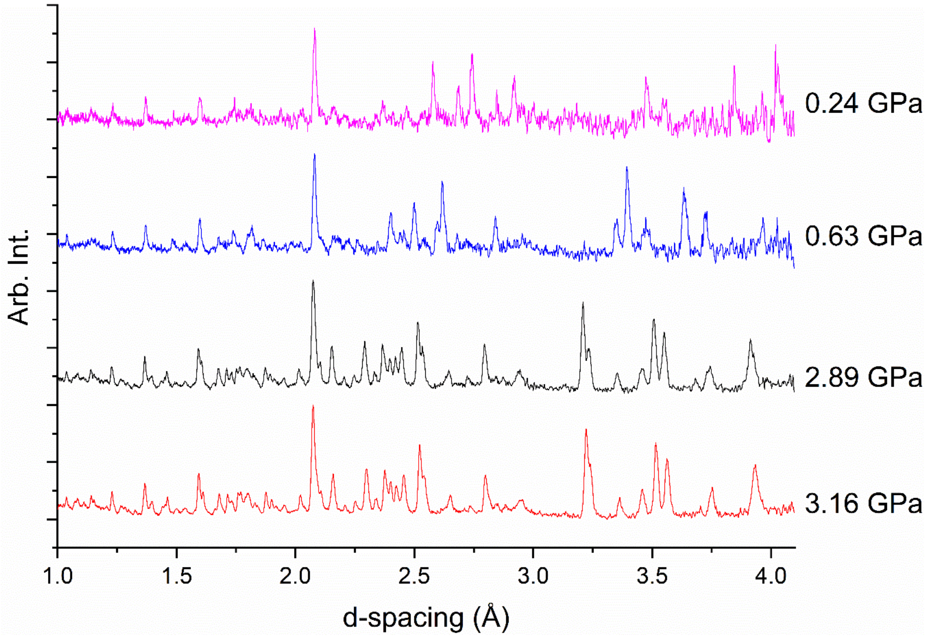


**Figure S2.** The decompression of Form II of caprolactam. At 0.63 GPa is a mixed phase of Form I and II and the transition to Form I is complete by 0.24 GPa. This was the lowest pressure achieved on decompression with the load taken off the sample. The sample was not left for any length of time so the pressure will not have had time to equilibrate.

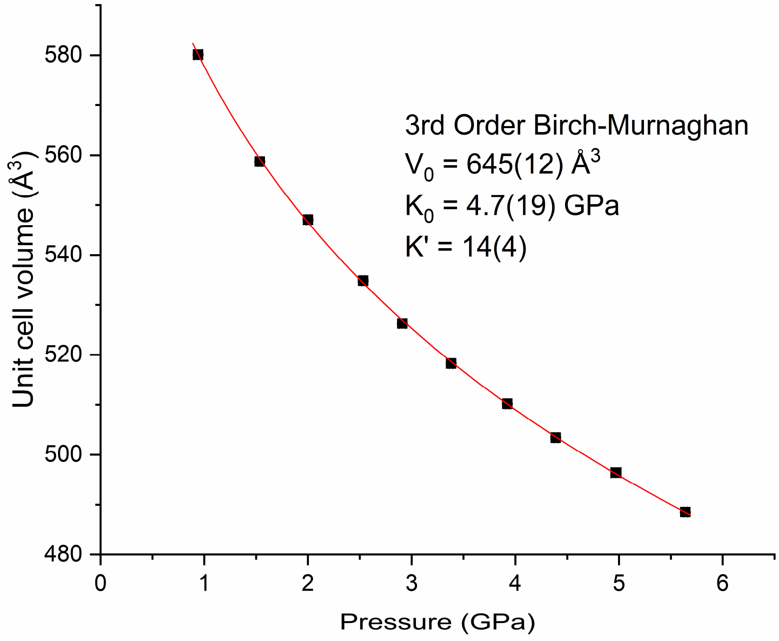


**Figure S3.** The 3rd Order Birch-Murnaghan Equation of State for Form III of caprolactam. Data points from the Pawley and Rietveld fits of the neutron diffraction data collected on the crystals obtained from ethanol-*d*6 and with perdeuterated pentane/isopentane as the pressure-transmitting medium.

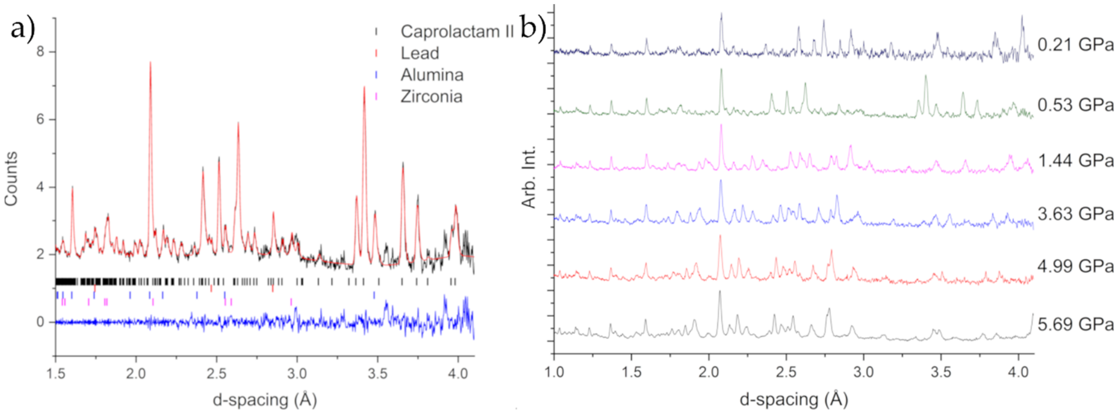


**Figure S4.** a) The Pawley fit of the data at 2 tonnes on decompression (0.53 GPa) using the cell parameters of Form III of caprolactam; b) the diffraction patterns of caprolactam on decompression from 5.69 GPa.

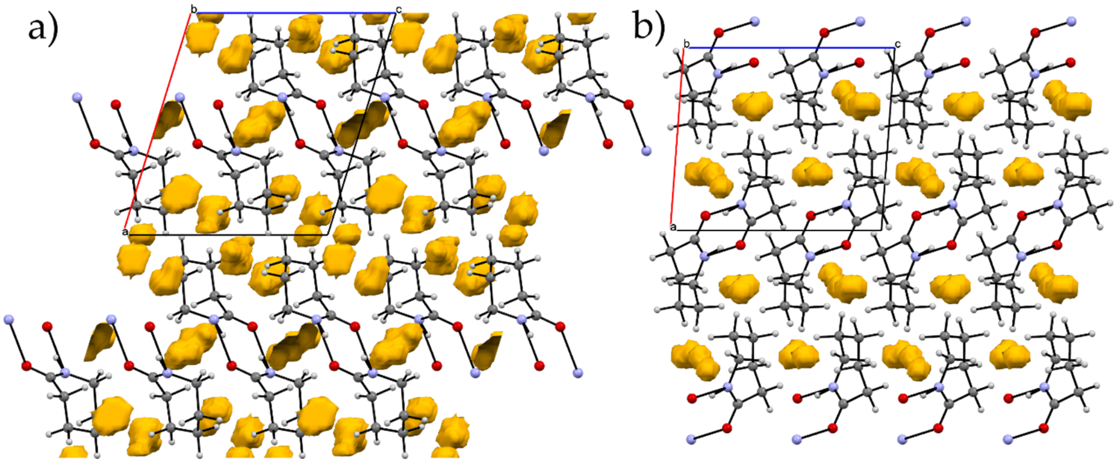


**Figure S5.** Void space using 0.5 Å probe radius and 0.2 grid spacing for a) Form II at 1.2 GPa and b) Form III at 0.94 GPa using Mercury [35] The percentage of void space in Form II was 4% of unit cell volume compared with 1.5% for Form III.

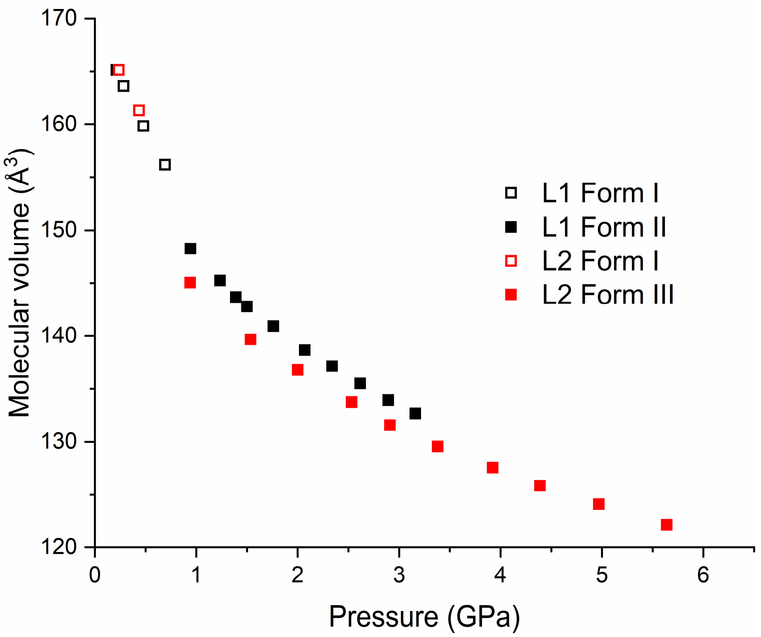


**Figure S6.** The molecular volume of each form as a function of pressure. The two loadings are color coded black (Loading 1; crystal from ethyl acetate) and red (Loading 2; crystal from ethanol-$d_6$). Hollow symbols represent Form I and solid symbols represent Forms II and III. Form III is consistently denser over the entire compression range.

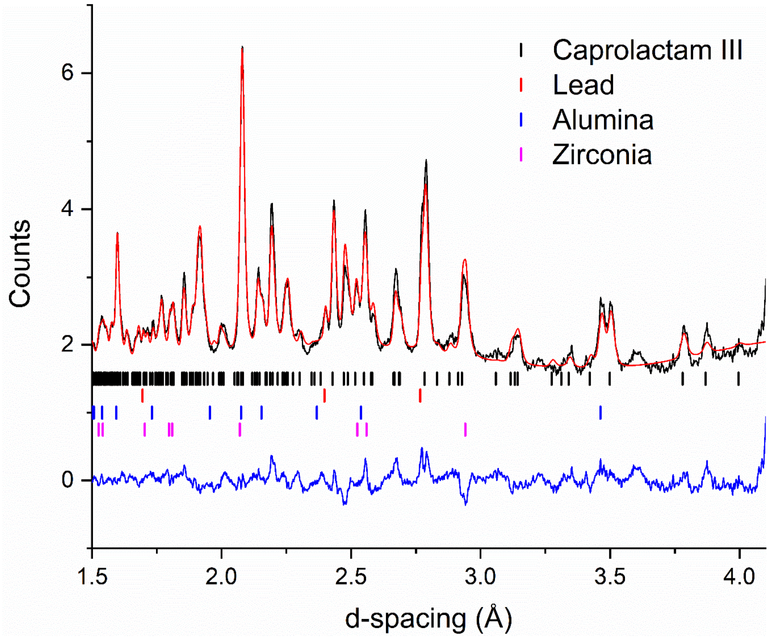


**Figure S7.** Rietveld fit of the 5.4 GPa data to Form III. The peak at 4.1 Å is due to the sample. The model used was taken from the geometry optimized structure and the orientation allowed to refine whilst keeping conformation of the molecule fixed.
